# Syringomatous adenoma of the nipple: a case report

**DOI:** 10.1186/s13256-015-0739-9

**Published:** 2015-11-13

**Authors:** Shoichi Ishikawa, Hirotaka Sako, Koji Masuda, Tomoko Tanaka, Kiyokazu Akioka, Yoshihiro Yamamoto, Yohei Hosokawa, Toshiaki Manabe

**Affiliations:** 1 Department of Surgery, Omihachiman Community Medical Center, 1379 Tsuchida-cho, Omihachiman City, Shiga 523-0082 Japan; 2 Department of Pathology and Laboratory Medicine, Omihachiman Community Medical Center, 1379 Tsuchida-cho, Omihachiman City, Shiga 523-0082 Japan; 3 Research Institute, Shiga Medical Center for Adults, 5-4-30, Moriyama, Moriyama City, Shiga 524-8524 Japan

**Keywords:** Metastasis, Nipple-sparing resection, Recurrence, Syringomatous adenoma of the nipple

## Abstract

**Introduction:**

Syringomatous adenoma of the nipple is a very rare benign tumor. To the best of our knowledge, there are no reports of a syringomatous adenoma of the nipple metastasizing, although these tumors are known to infiltrate locally and to recur if not totally resected.

**Case presentation:**

Our patient was a 41-year-old Japanese woman who complained of stiffness of her right nipple with abnormal discharge. Local resection of the tumor was performed. The pathological diagnosis was syringomatous adenoma of the nipple, and the resection margin was found to be positive. Accordingly, additional resection was recommended, but our patient did not allow another operation. After 1.5 years of careful follow-up, no local recurrence or distant metastasis has been observed.

**Conclusion:**

The optimal initial management of syringomatous adenoma of the nipple demands complete resection with histologically negative margins. However, from a cosmetic viewpoint, nipple-sparing resection could represent an alternative option for the treatment of syringomatous adenoma of the nipple.

## Introduction

Syringomatous adenoma of the nipple (SAN) is a very rare benign tumor that was first reported by Rosen in 1983 [1]. SAN shows locally infiltrating growth but does not metastasize [2]. Nonetheless, in some cases, this tumor may be misdiagnosed as a malignancy owing to its infiltrating growth pattern [3].

We present a rare case involving a 41-year-old woman with SAN, who underwent tumor resection with a histologically positive surgical margin, while sparing the nipple.

## Case presentation

**Fig. 1:**
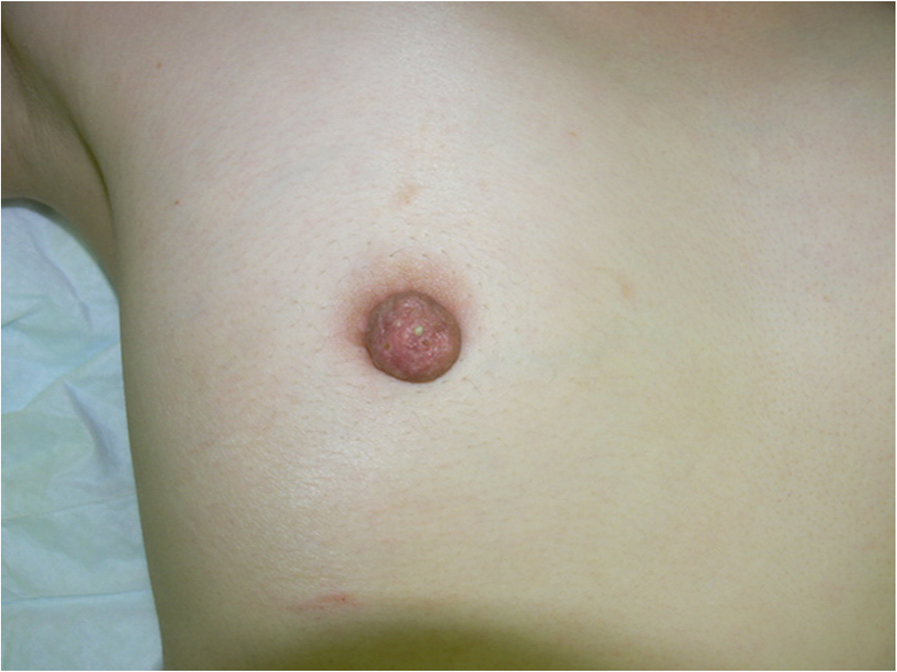
Appearance of the right nipple. The right nipple felt firm, but no mass was palpated. A milky secretion was noted. Our patient did not complain of pain, itching, or ulceration

A 41-year-old Japanese woman presented with firmness of her right nipple and abnormal nipple discharge (Fig. 1). The secretion was milky. She did not experience any pain, itching, or ulceration on the skin, and no lymph node swelling was detected at any site. No abnormality was seen in her left breast, and all standard laboratory test results were within the normal ranges.

**Fig. 2:**
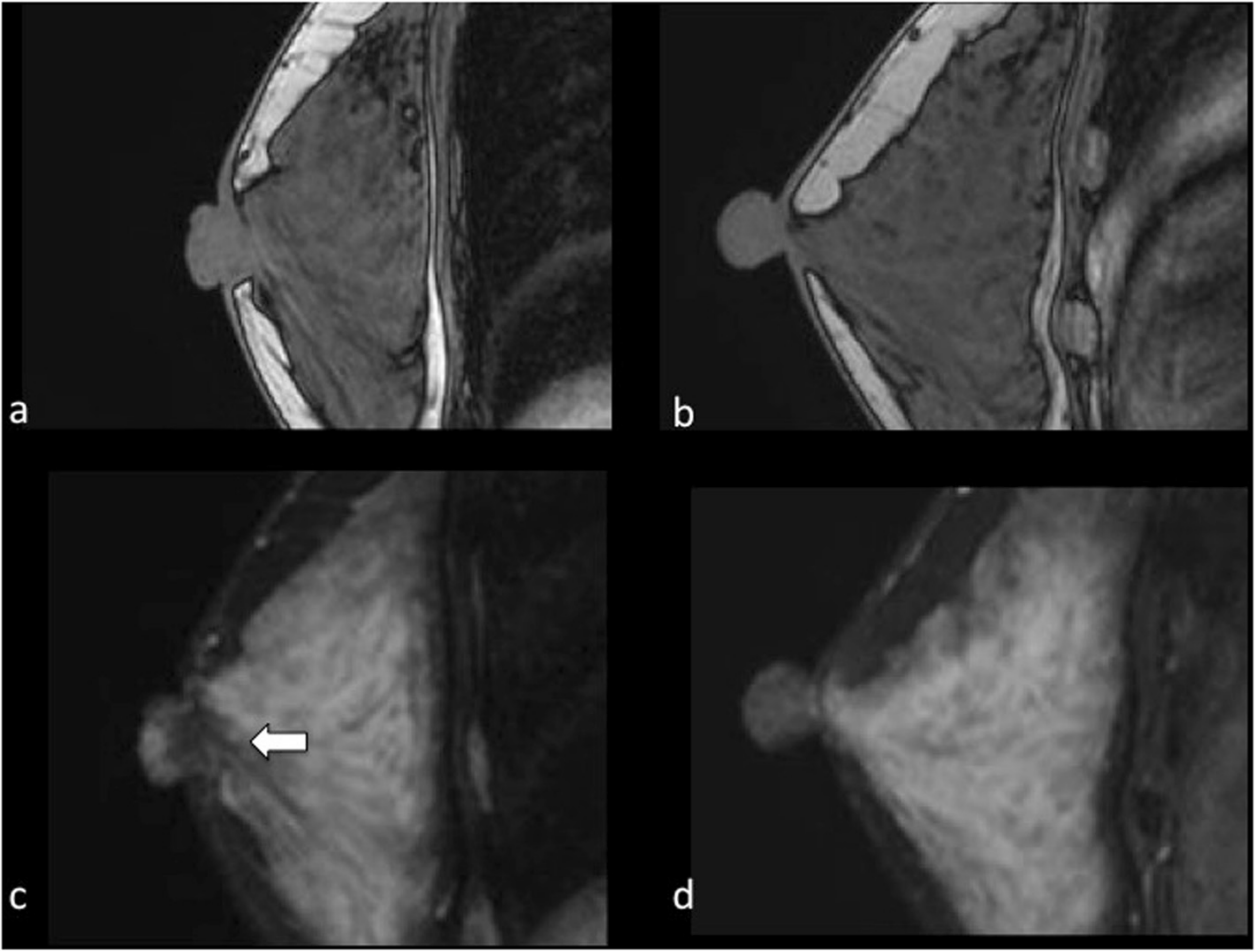
Magnetic resonance imaging. T1-weighted magnetic resonance imaging (MRI) did not show an obvious mass in the subareolar region of the right nipple (**a**). Conversely, T2-weighted MRI suggested a mass with low signal density (**c** *white arrow*). No mass was observed in the left side upon T1-weighted (**b**) or T2-weighted MRI (**d**)

No mass was observed upon mammography (MMG), although the subareolar region was more dense on the right side than the left. Moreover, foci of microcalcification were observed. Similarly, ultrasonography did not reveal an obvious mass. Magnetic resonance imaging (MRI) showed a mass with an irregular outline in the subareolar region of her right nipple. T1-weighted MRI did not show any obvious mass in her subareolar region, while a low signal density mass was observed on the T2-weighted images (Fig. 2). A cytodiagnosis of the nipple discharge did not indicate malignancy; however, the above-mentioned findings could not fully substantiate its benignancy. Hence, our patient consented to an incisional biopsy of her nipple lesion. A vertical incision was made to the right nipple skin along the Langer’s line, and a white, stiff mass with ambiguous borders was found in the subareolar area. As much of the tumor was removed as possible, while sparing her nipple.

**Fig. 3:**
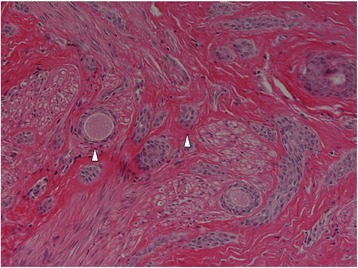
Micrograph of the tumor. Many of the proliferating ducts assuming a teardrop or comma-shaped configuration (*arrow heads*) were lined by double-layer or multiple-layer epithelial cells on microphotography. Squamoid solid nests are also seen. The tumor cells infiltrated the stroma between the smooth muscle bundles (hematoxylin and eosin; original magnification ×400)

The resected tumor was whitish, round, solid, and firm in consistency, and measured 7 mm in maximum diameter. With regards to the histopathology, the tumor was composed focally of ducts and tubules lined by a double layer of epithelial cells; the outer layer comprised small, cuboidal cells with scanty cytoplasm, while the inner layer consisted of rather flat cells with eosinophilic cytoplasm and centrally located small nuclei (Fig. 3). Many of the proliferating ducts assumed a teardrop or comma-shaped configuration. In addition, squamoid solid nests were found scattered among them, and the tumor cells infiltrated the stroma between the smooth muscle bundles. Immunohistochemical staining for Ki-67 indicated less than 5 % positivity. Although we were able to differentiate nipple duct adenoma, tubular carcinoma, and syringoma, the final pathological diagnosis was infiltrating syringomatous adenoma of the nipple. The clinical and histological features allowed us to exclude other lesions. The resection margin was found to be positive.

Because the tumor was so closely located to the epidermis of her nipple, additional resection of ample breast parenchyma and her right nipple was originally planned; however, our patient did not agree to immediate surgery. Although we explained the characteristics of the tumor and proposed additional resection with nipple preservation, our patient requested that surgery only be performed in case of a relapse.

Accordingly, she was kept under close surveillance. No local recurrence or distant metastasis was found during a 1.5-year follow-up period. We will continue careful monitoring for some years.

## Discussion

SAN is an extremely rare benign tumor. To the best of our knowledge, only 38 cases of SAN have been reported in the English literature [2, 4–6], including 36 female and 2 male cases. The age of these patients ranged from 11 to 87 years, with a mean age at presentation of 46.1 years. The resected tumors measured 5–40 mm in size, with a mean of 17.7 mm. Only two of these cases presented bilaterally [2, 6].

SANs commonly manifest as solitary firm masses in the subareolar or nipple region of the unilateral breast, and may also occur within the breast parenchyma. They may be either clinically asymptomatic, tender and painful on palpation, and/or present with itching and ulceration [7]. Nipple inversion or discharge is noted on occasion [8].

Upon MMG, SAN generally appears as a high-density mass in the subareolar region with an irregular outline, spicule formation, and microcalcification foci, while ultrasonography shows an ill-defined mass with heterogeneous internal echoes [9]. MRI can depict the mass more obviously than MMG. However, the imaging findings of SANs resemble those of malignant tumors, and, therefore, SAN may be indistinguishable from carcinoma on imaging examinations such as MMG, ultrasonography, and MRI [9].

Pathologically, the tumor grossly appears ill-defined, with firm-to-resilient consistency and a gray or white cut surface [1, 3]. Histologically, the lesion is composed of tubules, ductules, and strands of small, uniform, generally basophilic cells infiltrating the dermis of the surrounding skin and the stroma of the nipple [3]. Proliferating ducts, which are lined by a single or multiple layers of metaplastic squamous cells, may be present. These cell nests have a teardrop or comma-shaped appearance [10], and the tumor cells can infiltrate the stroma between the smooth muscle bundles, and even in the perineural region [9].

Nipple duct adenoma and well-differentiated tubular carcinoma are often confused with SAN. These entities, however, exhibit some distinctive characteristics. Nipple duct adenoma often ulcerates the skin, is usually better circumscribed, and does not invade the perineural region or smooth muscle bundles. Tubular carcinoma tends to occur deeper in the breast and is commonly located in the upper outer quadrant or away from the nipple. If tubular carcinoma extends to the nipple, it may cause nipple retraction and Paget’s disease [11]. Syringoma also exhibits clinical features that distinguish it from SAN, including presentation as a solitary lesion in the nipple [1].

Some researchers have reported the usefulness of immunohistochemical staining for p63 or S-100 protein [3, 6, 7]. However, in the present study we performed staining for Ki-67, a prognostic and predictive marker for breast cancer [12]. Cells express Ki-67 during the G_1_, S, G_2_, and M phases of the cell cycle, but not during the resting phase G_0_ [12]. Accordingly, high Ki-67 levels indicate that a tumor harbors a highly proliferative potential. In other words, Ki-67 staining helps to distinguish between benign and malignant tumors and to predict prognosis.

SAN has a tendency to show local recurrence when resected incompletely [6]. Accordingly, the optimal initial management demands complete resection with histologically negative margins [13]. If the margins appear involved, re-excision is recommended [3]. However, SAN often occurs in the dermis and subcutis regions of the nipple or areola [9]; in such cases, proper management necessitates total resection of the nipple–areolar complex. Some patients, especially young women, hope to retain the nipple. SAN is not a malignant tumor [1], and there have been no reports of SAN with distant metastasis [2]. If a patient requests preservation of the nipple, even with sufficient informed consent, tumor resection with nipple preservation should be considered. Nipple-sparing resection can yield excellent cosmetic results. If the tumor is so close to the nipple that nipple preservation is impossible, an appropriate treatment regimen should be selected for the patient. In such cases, however, careful postoperative monitoring is mandatory. Jones et al. reported times to recurrence ranging from 1.5 months to 4 years [11]. Accordingly, the follow-up duration should exceed 5 years if complete resection is not performed.

## Conclusion

We report the case of a 41-year-old woman with SAN. Considering her background and preferences, we resected the tumor while sparing her nipple. Considering the characteristics of the tumor, we cannot recommend nipple-sparing resection with positive margins. Although SAN may recur if not completely resected, SAN is a benign tumor and does not metastasize. With that in mind, from a cosmetic viewpoint, nipple-sparing resection can represent an alternative treatment option for SAN. However, the treatment regimen should be tailored to each patient. After nipple-sparing resection, careful and regular monitoring is necessary.

## Consent

Written informed consent was obtained from the patient for publication of this case report and accompanying images. A copy of the written consent is available for review by the Editor-in-Chief of this journal.
